# Antimicrobial Susceptibility and Molecular Features of Colonizing Isolates of *Pseudomonas aeruginosa* and the Report of a Novel Sequence Type (ST) 3910 from Thailand

**DOI:** 10.3390/antibiotics12010165

**Published:** 2023-01-12

**Authors:** Arnon Chukamnerd, Rattanaruji Pomwised, Sarunyou Chusri, Kamonnut Singkhamanan, Sanicha Chumtong, Kongpop Jeenkeawpiam, Chanida Sakunrang, Kuwanhusna Saroeng, Phanvasri Saengsuwan, Monwadee Wonglapsuwan, Komwit Surachat

**Affiliations:** 1 Division of Infectious Diseases, Department of Internal Medicine, Faculty of Medicine, Prince of Songkla University, Songkhla 90110, Thailand; 2 Division of Biological Science, Faculty of Science, Prince of Songkla University, Songkhla 90110, Thailand; 3 Department of Biomedical Sciences and Biomedical Engineering, Faculty of Medicine, Prince of Songkla University, Songkhla 90110, Thailand; 4 Division of Animal Production Innovation and Management, Faculty of Natural Resources, Prince of Songkla University, Songkhla 90110, Thailand; 5 Molecular Evolution and Computational Biology Research Unit, Faculty of Science, Prince of Songkla University, Songkhla 90110, Thailand; 6 Translational Medicine Research Center, Faculty of Medicine, Prince of Songkla University, Songkhla 90110, Thailand

**Keywords:** *Pseudomonas aeruginosa*, antimicrobial susceptibility, molecular feature, whole-genome sequencing, bioinformatics analysis, novel sequence type

## Abstract

*Pseudomonas aeruginosa* is an important pathogen as it can cause hospital-acquired infections. Additionally, it can also colonize in patients and in other various environments. Hence, this study aimed to investigate the antimicrobial susceptibility, and to study the molecular features, of colonizing isolates of *P. aeruginosa* from Songklanagarind Hospital, Thailand. Genomic DNA extraction, whole-genome sequencing (WGS), and bioinformatics analysis were performed in all studied isolates. The findings demonstrated that the majority of isolates were non-susceptible to colistin and carbapenem. For in silico study, multilocus sequence typing (MLST) revealed one novel sequence type (ST) 3910 and multiple defined STs. The isolates carried several antimicrobial resistance genes (*bla*_OXA-50_, *aph(3′)-IIb*, etc.) and virulence-associated genes (*fleN*, *waaA*, etc.). CRISPR-Cas sequences with different spacers and integrated bacteriophage sequences were also identified in these isolates. Very high SNPs were found in the alignments of the novel ST-3910 isolate with other isolates. A comparative genomic analysis exhibited phylogenetic clustering of our colonizing isolates with clinical isolates from many countries. Interestingly, ST-3981, ST-3982, ST-3983, ST-3984, ST-3985, ST-3986, ST-3986, ST-3986, ST-3987, and ST-3988, the new STs from published genomes, were assigned in this study. In conclusion, this WGS data might be useful for tracking the spread of *P. aeruginosa* colonizing isolates.

## 1. Introduction

*Pseudomonas aeruginosa* (*P. aeruginosa*) is a Gram-negative opportunistic bacterium that commonly infects and colonizes humans, animals, and plants, and it can be found in various environments, such as soil and water [1,2]. This bacterium is a member of the ESKAPEE pathogens, a group of antibiotic-resistant nosocomial pathogens, which includes *Enterococcus faecium*, *Staphylococcus aureus*, *Klebsiella pneumoniae*, *Acinetobacter baumannii*, *Pseudomonas aeruginosa*, *Enterobacter* spp., and *Escherichia coli* [3]. The Centers for Disease Control and Prevention (CDC) reported on the incidence of multidrug-resistant (MDR) *P. aeruginosa* in American hospitalized patients in 2017. This report outlined the causes of a variety of hospital-acquired infections, including respiratory tract infections (RTIs), bloodstream infections (BIs), urinary tract infections (UTIs), and surgical site infections (SSIs); all of which are considered to have a serious threat level [4]. In this report, there were an estimated 32,600 cases, with 2700 estimated deaths, and $767,000,000 estimated attributable healthcare costs in the United States of America (USA) [4]. Additionally, infection and colonization by *P. aeruginosa*, particularly MDR strains, are classified as the third group of Gram-negative pathogens in hospitals within Thailand [5].

Over the past decade, multiple studies have reported on the whole-genome sequencing (WGS) data of *P. aeruginasa* from several regions. In the study of Quick et al. (2014), WGS and bioinformatics analysis were conducted for 141 *P. aeruginosa* isolates from patients and contaminated sources (hospital water and the ward environment) [6]. The phylogenetic tree showed eight clades (A to H), which contained many clades of clinical isolates and a single clade of environmental isolates. The single nucleotide polymorphisms (SNPs) in the genes associated with intrinsic resistance mechanisms (mutations) to antimicrobial agents were identified in the studied isolates. Cottalorda et al. (2021) also sequenced 108 *P. aeruginosa* urinary isolates obtained from two to five samples of seven hospitalized patients in a French hospital [7]. Their results demonstrated that a single clone type of *P. aeruginosa* colonized in each patient, which was considered by <6000 SNPs between a pair of the isolates. Another study by Zhu et al. (2021) reported the WGS data of 151 carbapenem-resistant *P. aeruginosa* from China [8]. Eleven defined STs that included ST-463 as the most frequent and one undefined ST were found, and eight plasmid types with 13 plasmid patterns were observed. In the study in Thailand, Cazares et al. (2020) performed WGS in 23 of 48 MDR *P. aeruginosa* clinical isolates at a teaching hospital at Mahidol University, Bangkok, Central Thailand [9]. In this study, the majority of the isolates belonged to the dominant ST-253 (*n* = 3) in addition to many other STs (e.g., ST-233, ST-244, ST-270, ST-357, ST-491, ST-708, ST-1330, and so on); however, some isolates could not be assigned to the STs. Additionally, Soonthornsit et al. (2022) sequenced 10 antimicrobial-resistant *P. aeruginosa* isolates from a veterinary teaching hospital in Nakhon Pathom, Central Thailand [10]. They also found a high-risk clone ST-235 (*n* = 3) as well as other STs (ST-244, ST-485, ST-606, and ST-3405). Moreover, the multilocus sequence typing (MLST) and SNP analysis exhibited a correlation between the isolates from ward rooms to those from operating, wound care, and examination rooms. Even though these studies have reported genomic insights into *P. aeruginosa*, there is still a lack of data on whole-genome sequences of *P. aeruginosa*, especially the colonizing isolates from Southern Thailand.

Hence, the objectives of this study were to evaluate antimicrobial susceptibility and to study the molecular characteristics of *P. aeruginosa* isolates colonized in the patients at the Medicine Ward, Songklanagarind Hospital, Southern Thailand.

## 2. Results

### 2.1. Antimicrobial Susceptibility Profiles

Antimicrobial susceptibility profiles in 13 colonizing isolates of *P. aeruginosa* showed that all isolates were susceptible to almost all tested antimicrobial agents, including piperacillin-tazobactam, ceftolozane-tazobactam, amikacin, gentamicin, tobramycin, ciprofloxacin, and levofloxacin (Table 1). Meanwhile, a total of nine, three, two, and one isolates were resistant to colistin with minimum inhibitory concentrations (MICs) ranging from 4 to 8 µg/mL, imipenem with MICs ranging from 16 to 32 µg/mL, meropenem with MICs ranging from 8 to 16 µg/mL, and doripenem with MIC at 8 µg/mL, respectively. The results also exhibited that all (*n* = 13) and over a half (*n* = 9) of the isolates were non-susceptible (intermediate to resistant) to colistin and carbapenems, respectively.

### 2.2. Genome Assembly Quality

The genome assembly quality of all the studied isolates is demonstrated in Table S1 and Figure S1. In the QUAST results, the genome lengths of 6,202,244 to 6,974,963 bps were obtained from 13 *P. aeruginosa* isolates, and N50 ranged from 391,215 to 931,346 bps. A total of 62 to 266 contigs were generated from these isolates, and L50 ranged from three to seven contigs. In the BUSCO assessment results, even though one duplicated gene and a few fragmented genes were detected in our *P. aeruginosa* genomes, very high completeness without missing genes was found in all the genomes.

### 2.3. Sequence Types and Serotypes

Multilocus sequence typing (MLST) was performed in all colonizing isolates of *P. aeruginosa* to identify the sequence types (STs). The results revealed that two isolates belonged to ST-162, while 10 isolates belonged to different STs (ST-266, ST-270, ST-313, ST-500, ST-532, ST-647, ST-980, ST-1097, ST-1197, and ST-1240), as presented in Table 2. Notably, the PA02 isolate was not assigned to any ST due to its new allelic profile. Afterward, the assembled genome of the PA02 isolate was submitted with a new MLST profile into PubMLST (https://pubmlst.org/, accessed on 28 April 2022), and ST-3910 was then released for the PA02 isolate. In the prediction of *P. aeruginosa* serotypes, the results revealed that O11, O6, O3, O1, O5, and O10 were identified in four, four, two, one, one, and one isolates, respectively (Table 2).

### 2.4. Acquired Antimicrobial Resistance Genes

**Figure 1:**
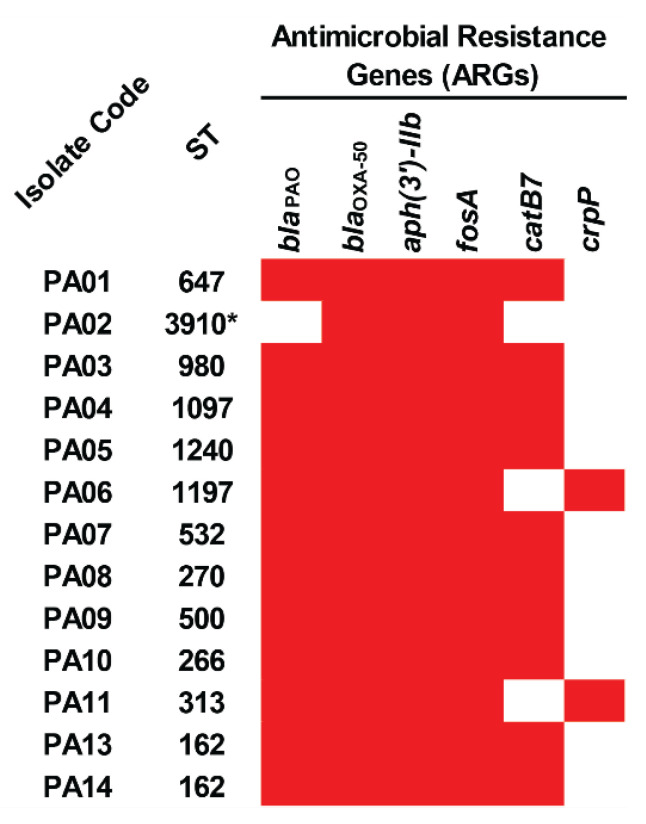
Distribution of antimicrobial resistance genes (ARGs) in the colonizing isolates of *P. aeruginosa*. The red box represents the gene presence. ST, sequence type. * Novel ST.

In the detection of acquired antimicrobial resistance genes (ARGs), it was found that all the colonizing isolates of *P. aeruginosa* carried *bla*_OXA-50_, *aph(3′)-IIb*, and *fosA*, while 12, 10, and two isolates harbored *bla*_PAO_, *catB7*, and *crpP* genes (Figure 1 and Table S2). Among the studied isolates, a novel ST-3910 (PA02) possessed only three ARGs, including *bla*_OXA-50_, *aph(3′)-IIb*, and *fosA*. These genes may provide resistance to β-lactam (*bla*_OXA-50_ and *bla*_PAO_), aminoglycoside (*aph(3′)-IIb*), fosfomycin (*fosA*), chloramphenicol (*catB7*), and ciprofloxacin (*crpP*).

### 2.5. Insertion Sequence Elements

**Figure 2:**
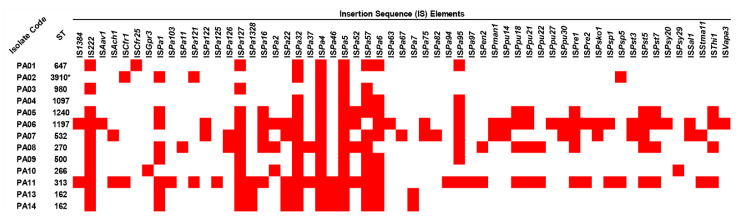
Distribution of insertion sequence (IS) elements in the colonizing isolates of *P. aeruginosa*. The red box represents the gene presence. ST, sequence type. * Novel ST.

To look for mobile genetic elements (MGEs), insertion sequence (IS) elements and integrons in all colonizing isolates of *P. aeruginosa* were identified. The results showed that 57 IS elements were present in the isolates. Among them, IS*Pa4* and IS*Pa5* were detected in all isolates, while IS*Pa32*, IS*222*, IS*Pa127*, IS*Pa57*, and IS*Pa6* were found in 12, 11, 11, 11, and 10, respectively (Figure 2 and Table S3). In contrast, IS*Aav1*, IS*Cfr25*, IS*Gpr3*, IS*Pa103*, IS*Pa11*, IS*Pa125*, IS*Pa63*, IS*Pa67*, IS*Pa82*, IS*Pa94*, IS*Pa97*, IS*Ppu27*, IS*Psy20*, IS*Psy29*, and IS*Vapa3* were only found in one isolate. Among these isolates, the ST-313 (PA11) isolate possessed the highest number of IS elements, followed by ST-1197 (PA06), ST-532 (PA07), ST270 (PA08), and so on. However, via in silico analysis, integrons were not found in the studied isolates.

### 2.6. Virulence-Associated Genes

**Figure 3:**
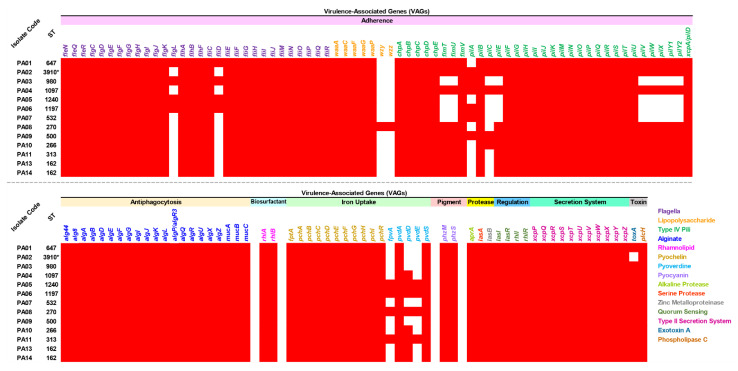
Distribution of virulence-associated genes (VAGs) in the colonizing isolates of *P. aeruginosa*. The red box represents the gene presence. ST, sequence type. * Novel ST.

Virulence-associated genes (VAGs) were explored in all the colonizing isolates of *P. aeruginosa*, which possessed 115 to 127 genes encoding several virulence factors, as shown in Figure 3 and Table S4. All isolates carried all detected genes encoding antiphagocytosis (alginate), biosurfactant (rhamnolipid), iron uptake (pyochelin), pigment (pyocyanin), protease (alkaline protease, serine protease, and zinc metalloproteinase), regulation (quorum sensing), secretion system (type II secretion system), and toxin (phospholipase c). Furthermore, high numbers of genes encoding adherence (flagella, lipopolysaccharide, and type IV pili) were found in all isolates. A gene encoding exotoxin A (*toxA*) was also present in all isolates, except for the novel ST-3910 (PA02) isolate. On the other hand, among the genes encoding lipopolysaccharide, only the ST-270 (PA08) isolate carried *wzy* and *wzz* genes.

### 2.7. Bacteriocins

**Figure 4:**
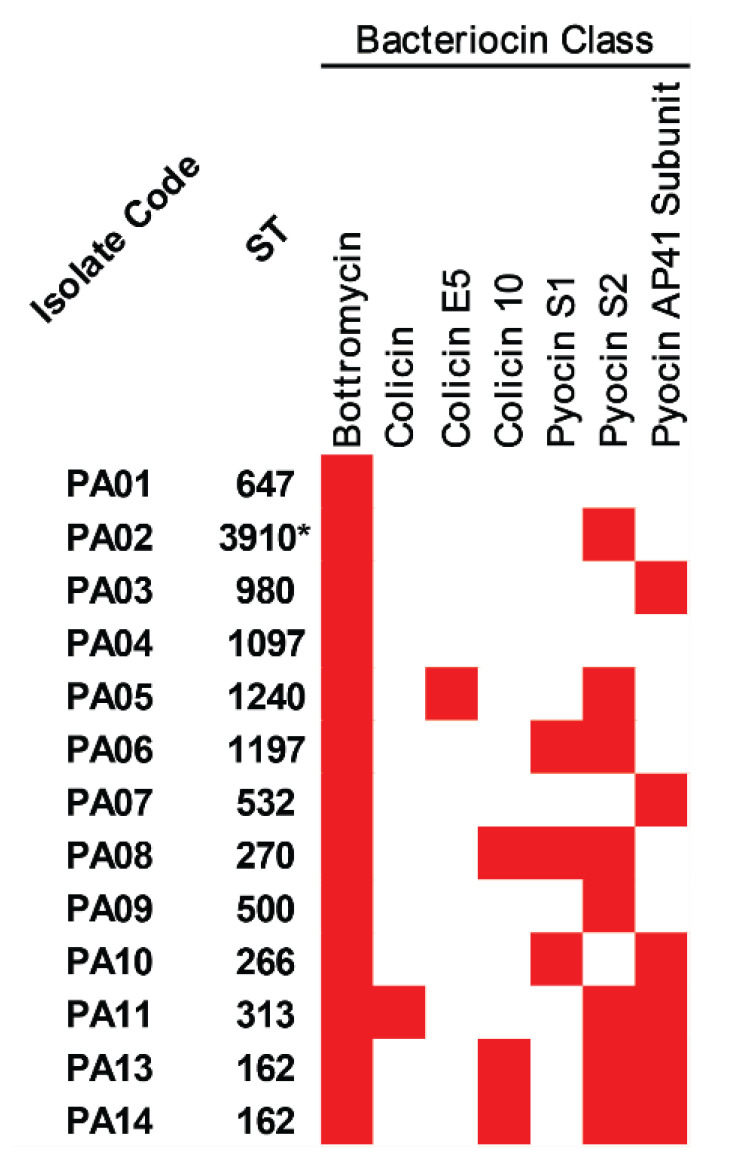
Bacteriocins in the colonizing isolates of *P. aeruginosa*. The red box represents the gene presence. * Novel ST.

With regards to bacterial competition, bacteriocin-encoding genes were predicted in all colonizing isolates of *P. aeruginosa*, and the results are illustrated in Figure 4 and Table S5. Bottromycin and pyocin AP41 subunit were positive in all and eight isolates, respectively, while colicin 10 and pyocin S1 were found in three isolates. In addition, colicin and colicin E5 were only detected in the ST-313 (PA11) and ST-1240 (PA05) isolates, respectively.

### 2.8. CRISPR-Cas System

To evaluate the bacterial defense system, a clustered, regularly interspaced, short palindromic repeats and CRISPR-associated protein (CRISPR-Cas) system was explored in all colonizing isolates of *P. aeruginosa*. We found the CRISPR-Cas regions in seven isolates, as listed in Table 3 and Table S6. Even though the Cas region was not detected in the ST-266 (PA10) isolate, one CRISPR region carrying nine spacer sequences was detected in this isolate. Remarkably, nine CRISPR loci with four to twenty-seven different spacer sequences and two Cas types (IF and IE) were harbored by the novel ST-3910 (PA12) isolate. Two groups of the *cas* genes (*cas1*_*cas3-cas2*_*csy1*_*csy2*_*csy3*_*cas6* and *cas6*_*csy3*_*csy2*_ *csy1*_*cas3-cas2*_*cas1*) were classified as Cas type IF, while the other two groups (*cas2*_*cas1*_*cas6*_*cas5*_*cas7*_*cse2*_*cse1*_*cas3* and *cas3*_*cse1*_*cse2*_*cas7*_*cas5*_*cas6*_*cas1*_*cas2*) were predicted as Cas type IE.

### 2.9. Integrated Bacteriophage Genomes

**Figure 5:**
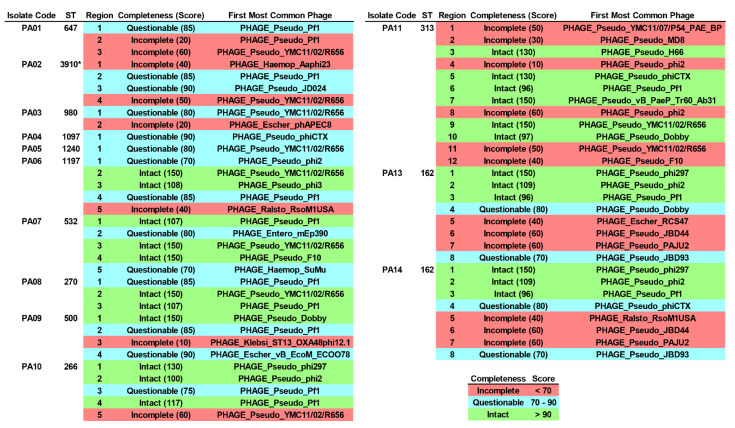
Integrated bacteriophage genomes (IBGs) in the colonizing isolates of *P. aeruginosa*. ST, sequence type. * Novel ST.

Lysogenic bacteriophage infection is one of the factors for driving genes (e.g., ARGs, VAGs, etc.) to other bacterial cells. Here, integrated bacteriophage genomes (IBGs) in all the colonizing isolates of *P. aeruginosa* were screened for. At least a part of the bacteriophage sequences had been integrated into the genomes of the studied isolates, as shown in Figure 5 and Table S7. A total of 23, 20, and 18 regions were identified as intact, incomplete, and questionable, respectively. A majority of those regions were classified as *Pseudomonas* phages (*n* = 52), while some regions were classified as *Escherichia* phages (*n* = 3), *Haemophilus* phages (*n* = 2), *Ralstonia* phages (*n* = 2), an *Enterobacter* phage (*n* = 1), and a *Klebsiella* phage (*n* = 1). The partial sequence of PHAGE_Pseudo_Pf1 (*n* = 13) was dominantly found in the isolates, followed by PHAGE_Pseudo_YMC11/02/R656 (*n* = 10), PHAGE_Pseudo_phi2 (*n* = 6), PHAGE_Pseudo_phiCTX (*n* = 3), PHAGE_Pseudo_Dobby (*n* = 3), PHAGE_Pseudo_phi297 (*n* = 3), PHAGE_Pseudo_phiCTX (*n* = 3), and so on. Most of the sequences were encoded for bacteriophage composition proteins (head/capsid, tail, and plate) as well as bacteriophage enzymes (integrase, terminase, recombinase, and transposase).

### 2.10. Genomic Diversity and Phylogenetic Relationship

**Figure 6:**
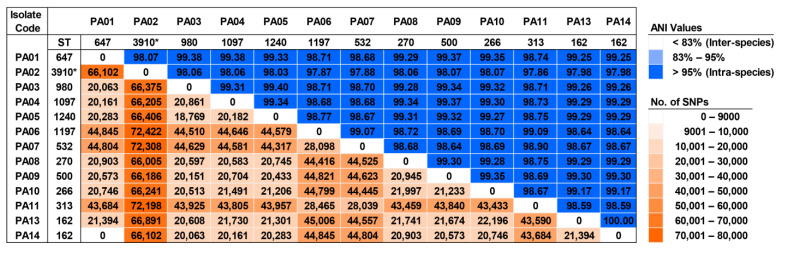
The matrix of pairwise average nucleotide identity (ANI) values and pairwise single nucleotide polymorphism (SNP) distance among the colonizing isolates of *P. aeruginosa*. ST, sequence type. * Novel ST.

To study the genomic diversity of the studied isolates, pairwise average nucleotide identity (ANI) and pairwise single nucleotide polymorphism (SNP) distance were analyzed, and these results are demonstrated in Figure 6. The ANI values ranged from 97.86–100%, showing that all colonizing isolates were intra-species of *P. aeruginosa*. An ANI value of 100% was only found in an alignment of the ST-162 genomes (PA13 and PA14). Although a total of 18,769 to 72,422 SNPs were found in the pairs of these sequences, none of the SNPs were identified in an alignment between the ST-647 (PA01) and ST-162 (PA14) genomes. On the contrary, 21,394 SNPs were detected in a pair of ST-162 sequences (PA13 and PA14). Over 66,000 SNPs were predominantly observed in the pairs of the novel ST-3910 (PA02) sequences with other sequences.

**Figure 7:**
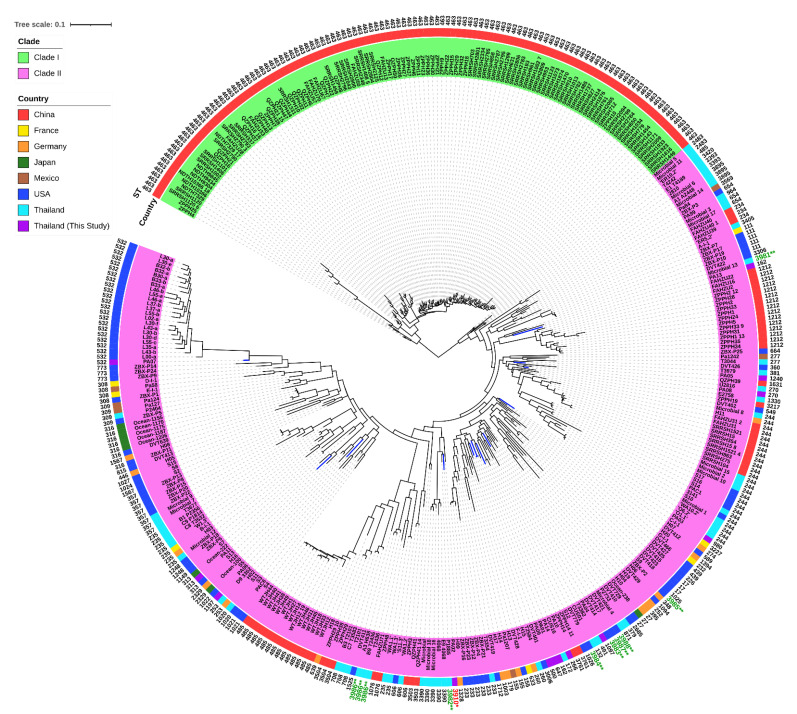
A single nucleotide polymorphism (SNP) phylogenetic tree of our genomes (blue linage) and other available genomes of *P. aeruginosa*. ST, sequence type. * Novel ST from our *P. aeruginosa* genome (red text). ** Novel STs from previously published genomes of *P. aeruginosa* (green text).

In addition, our genomes (*n* = 13) were compared with previously published genomes (*n* = 357) of *P. aeruginosa* clinical isolates, and the metadata of the included genomes is illustrated in Table S8. The pan-genome profiles of 370 genomes showed 35,430 pan genes, which included 4004 (11.30%) core genes and 31,426 (88.70%) accessory genes (Figure S2 and Table S9). Many genes encode for hypocritical proteins. Interestingly, the ST-463 isolates in the upper monophyletic group (clade I) harbored 38 unique genes. These genes encode for hypothetical proteins (*n* = 29) and other identified proteins (*n* = 9), including tyrosine recombinase XerC, HTH-type transcriptional repressor GlaR, putative NADH-specific resorcinol 4-hydroxylase, hexuronate transporter, ATP-dependent DNA helicase Rep, ATP-dependent RNA helicase RhlE, RNA polymerase-associated protein RapA, GTPase Era, and ADP-ribosylarginine hydrolase Tri1. More importantly, 127 genes-encoding unique proteins, including hypothetical proteins (*n* = 109) and other identified proteins (*n* = 18), had the highest frequency that exited in the novel ST-3910 (PA02) isolate, compared with the other 12 genomes of our colonizing isolates. The SNP-based phylogenetic tree of 370 genomes revealed two major clades (Figure 7), which is similar to the pan-genome matrix against the phylogenetic tree based on accessory genes (Figure S2). Clade I contained only the ST-463 isolates (*n* = 116) from China, while clade II included multiple isolates (*n* = 254) belonging to 99 different STs from various countries. Our colonizing isolates of *P. aeruginosa* were distributionally found in several subclades of clade II. Besides this, a total of 10 published genomes from other studies in Thailand (*n* = 5) and the USA (*n* = 5) could not be assigned with STs. These genomes were then submitted into PubMLST, and 10 novel STs (ST-3981, ST-3982, ST-3983, ST-3984, ST-3985, ST-3986, ST-3986, ST-3986, ST-3987, and ST-3988) were assigned accordingly. Notably, some published genomes from other areas of Thailand as well as our genomes were clustered in different subclades.

## 3. Discussion

*P. aeruginosa*, especially antibiotic-resistant strains, are categorized as the third pathogen of Gram-negative bacteria causing nosocomial infections in Thailand [5]. Importantly, multidrug-resistant *P. aeruginosa* is considered as a serious threat level by the Centers for Disease Control and Prevention (CDC), 2019 [4]. In addition to the infections, colonization by this pathogen is raising concerns and challenges in controlling the spread of *P. aeruginosa*, both within the same ward or different wards, as well as intra- or inter-hospitals.

The results from this study revealed that 12 colonizing isolates of *P. aeruginosa* belonged to 11 defined STs with ST-162 being the most prevalent; however, the novel ST-3910 was assigned for the PA02 isolate. Among the defined STs, ST-270 was similarly found in a previous study from the central part of Thailand [9]. The antimicrobial susceptibility results demonstrated that the colonizing isolates were still susceptible to many selected antibiotics. Certain isolates were resistant to colistin and carbapenem (imipenem, meropenem, and/or doripenem). In WGS analysis, the detection of ARGs showed that the aminoglycoside resistance gene (*aph(3′)-IIb*) was present in all colonizing isolates of *P. aeruginosa*. In contrast, the isolates were phenotypically susceptible to all selected aminoglycosides (amikacin, gentamicin, and tobramycin). It was therefore hypothesized that the *aph(3′)-IIb* gene carried by these isolates might be truncated and cannot be expressed, leading to aminoglycoside susceptibility. Furthermore, three ARG patterns were detected in the isolates and the novel ST-3910 (PA02) harbored the lowest number of ARGs. All the ARGs detected in this study are commonly found in *P. aeruginosa* clinical isolates, especially the *bla*_OXA-50_ and *bla*_PAO_ genes [11,12,13].

In the prediction of factors causing genetic movements, such as MGEs, insertion sequences IS*Pa4* and IS*Pa5* were identified in all studied isolates. IS*Pa4* and IS*Pa5* are unclassified IS elements that were first detected with lengths of 2564 and 965 bps, respectively, in the mucoid isolates of *P. aeruginosa* PAO-muc (accession number U16785) collected from cystic fibrosis patients [14]. A previous study showed that these IS elements were present together and might have originated from plasmids and/or insertion sequences [14]. Moreover, 500 bp of both IS elements contained 94% similarity with IS*Pa6*; additionally, these three IS elements were located upstream of the exotoxin A-encoding gene (*toxA*) [14,15,16]. Nevertheless, we found that although all the studied isolates possessed IS*Pa4* and IS*Pa5*, the *toxA* gene was harbored by almost all isolates, except for the novel ST-3910 (PA02) isolate. Thus, these findings are not consistent with the phenomenon from earlier studies [14,15,16].

Besides the *toxA* gene, many other VAGs were also observed in at least four studied isolates. This was with the exception of the *wzy* and *wzz* genes-encoding lipopolysaccharide, which generally plays a role in adherence, as this was only found in the ST-270 (PA08) isolate. The Wzy–Wzz interaction has been previously reported, and these two proteins were associated with B-band LPS synthesis in *P. aeruginosa* [17,18]. Our isolates contained nine major virulence factors, including adherence, antiphagocytosis, biosurfactant, iron uptake, pigment, protease, regulation, secretion system, and toxin. These findings indicate a high pathogenicity of the colonizing isolates of *P. aeruginosa*, which may contribute to acute and chronic infections [19,20]. In the prediction of bacterial competition, the studied isolates contained at least one bacteriocin-encoding gene being found. The bottromycin detected in all isolates has an activity to inhibit aminoacyl tRNA in the connection with the A site on the 50S ribosome in DNA replication. Prior studies reported that bottromycin has antibacterial activity combatting vancomycin-resistant Enterococci (VRE) and methicillin-resistant *Staphylococcus aureus* (MRSA) [21,22].

In addition, CRISPR-Cas regions, the bacterial adaptive immune system, were identified in the studied isolates. These findings revealed type IF and/or IE CRISPR-Cas systems in seven isolates. Among them, the novel ST-3910 (PA12) isolate harbored the highest number of CRISPR loci, with many distinct spacers and two Cas types. This could confirm their evolution against invaded foreign genetic elements from bacteriophage infections and/or external plasmids [23]. Moreover, the evidence of bacteriophage infections was also assessed. The findings demonstrated various bacteriophage sequences encoding composition proteins as well as significant enzymes, and most of them were identified from *Pseudomonas* phages, especially *P. aeruginosa* filamentous 1 (Pf1) phage (PHAGE_Pseudo_Pf1). The Pf1 phage has been reported as one of the key factors that promote the pathogenicity of *P. aeruginosa*, including biofilm formation and antiphagocytosis [24]. It was therefore speculated that our colonizing isolates have been infected with lysogenic bacteriophages and these viruses might be the important factors driving genetic materials, the so-called horizontal gene transfers (HGTs), from infecting to colonizing isolates, resulting in the increased virulence in the colonizing isolates.

To study the genomic diversity, a comparative genomic analysis was performed. All pairwise ANI values exhibited intra-species of *P. aeruginosa*. Meanwhile, pairwise SNP distances showed different numbers in almost all pairs of the alignments, and high SNPs were found in the pairs of the novel ST-3910 (PA02) with other isolates. Concerning the PA13 and PA14 isolates, although they belonged to the same ST (ST-162) and their genomic features were similar with an ANI value of 100%, a total of 21,394 SNPs were identified and these were clustered in the distinct subclades within the clade II (Figure 6 and Figure 7). Thus, it was speculated that many recombination events might have occurred in other parts within the core genomes of these isolates, not in the seven housekeeping genes used in MLST, leading to a high SNP distance [25]. On the other hand, although the PA01 and PA14 isolates belonged to different STs (ST-647 and ST-162, respectively) and their genomic features were different with the ANI value of 99.25%, none of the core genome SNP distances were observed and they were grouped in the same subclade within clade II (Figure 6 and Figure 7). It was hypothesized that this may be the opposite of a previously mentioned case (PA13 and PA14, the ST-162 isolates). The core genome of the PA01 isolate was probably identical to that of the PA14 isolate, whereas some SNPs should be found in the MLST alleles that resulted in defining distinct STs. Ambiguous nucleotides and dashes might be found in the short-read WGS data of these isolates, which is not included in the running of the SNP-dists program, leading to an SNP distance of zero.

In comparison with the published genomes of the clinical isolates from several countries, these findings revealed a high level of accessory genes, indicating an open pan-genome and extensive genomic diversity of *P. aeruginosa*. Nevertheless, genes encoding unique and specific proteins in our genomes of colonizing isolates were not observed, compared with previously published genomes of infecting isolates. Additionally, the SNP-based phylogenetic tree demonstrated that our genomes and other published genomes were similarly clustered within the phylogenetic clade II. This phenomenon indicated a close genomic relatedness within the colonizing and infecting isolates.

In comparison with previous studies, some ARG, VAGs, IS elements, and IBGs patterns of the genomes of *P. aeruginosa*, carbapenem-resistant *Acinetobacter baumanii* (CRAB), and carbapenem-resistant Enterobacterales (CRE) were different [26,27,28,29,30]. For example, in the detection of β-lactam resistance genes, the *bla*_OXA-23_, *bla*_NDM_, and *bla*_PAO_ genes were only found in CRAB, CRE, and *P. aeruginosa*, respectively. These findings confirmed that the acquisition of specific genes is dependent on bacterial species. Furthermore, the studies of IBGs in different bacterial species could confirm the narrow/specific host range of bacteriophages.

Finally, even though the genomic insights into the draft genomes of *P. aeruginosa* colonizing isolates were analyzed, further experiments may be required in the future. For example, one limitation of this study was that the complete plasmids from short-read WGS data could not be identified. Therefore, long-read WGS data would be beneficial to identify both classified and unclassified plasmids as well as integrons. Identifying ARGs and VAGs on the plasmids and/or integrons can confirm HGTs, which can track the spread of these particular genes among *P. aeruginosa* and other Gram-negative bacteria. Importantly, complete genomes by long-read sequencing would be used as the reference genomes of *P. aeruginosa* colonizing isolates.

## 4. Materials and Methods

### 4.1. Colonizing Isolates of Pseudomonas aeruginosa

In 2017, a total of 13 *P. aeruginosa* isolates were collected from patients, admitted to the Medicine Ward of Songklanagarind Hospital, Songkhla, Thailand, who were suffering from various underlying diseases, except for *P. aeruginosa* infection. The species of *P. aeruginosa* was identified by biochemical testing and confirmed by 16S rRNA amplicon sequencing. Among 13 isolates, 6 and 7 isolates were obtained from the rectum and the throats of patients, respectively.

### 4.2. Antimicrobial Susceptibility Testing

The susceptibility of *P. aeruginosa* to 11 antimicrobial agents (piperacillin-tazobactam, ceftolozane-tazobactam, doripenem, imipenem, meropenem, colistin, gentamicin, tobramycin, amikacin, ciprofloxacin, and levofloxacin) was evaluated by the broth microdilution method, following Clinical and Laboratory Standard Institute (CLSI) 2018 guidelines [31]. Briefly, the 2-fold serial concentration of antimicrobial agent was prepared in a 96-well culture plate. Afterward, the *P. aeruginosa* culture was adjusted to a 0.5 MacFarland standard and further diluted to 1:100. The desired concentration of *P. aeruginosa* was added to a 96-well culture plate containing serial dilution of antimicrobial agents and they were incubated at 37 °C for 18 h. The results were measured using resazurin dye and interpreted according to the CLSI breakpoint. *P. aeruginosa* ATCC 27853 and *Escherichia coli* ATCC 25922 were used as quality controls.

### 4.3. DNA Extraction and Whole-Genome Sequencing

Genomic DNA (gDNA) of 13 *P. aeruginosa* isolates was extracted using GF-1 Bacterial DNA Extraction Kit, following the manufacturer’s instructions. The concentration and purity of extracted gDNA were checked using Thermo Scientific NanoDrop 2000/2000c Spectrophotometers and agarose gel electrophoresis. The qualified DNA sample was sent to perform short-read whole-genome sequencing (WGS) with the MGISEQ-2000 (MGI, Shenzhen, China).

### 4.4. Genome Assembly and Annotation

The quality of sequence reads was initially assessed by MGI Tech Co. Ltd. The qualified sequence reads were de novo assembled using Unicycler v0.4.7 [32]. The quality and completeness of assembled genomes were investigated using QUAST v4.0 [33] and BUSCO v5.2.2 [34], respectively. The contaminant sequences were evaluated and removed using Kraken2 v2.0.7 [35] and Geneious R10.26 [36]. Then, the assembled genome was annotated using Prokka v1.12 [37].

### 4.5. Sequence Analysis

The sequence types (STs) and acquired antimicrobial resistance genes (ARGs) were identified using staramr v0.7.2 (https://github.com/phac-nml/staramr, accessed on 1 October 2022) [38,39,40]. Serotypes of *P. aeruginosa* were predicted using *Pseudomonas aeruginosa* serotyper (PAst) 1.0 in the center for genomic epidemiology (CGE) [39,41]. Virulence-associated genes (VAGs) were investigated using BLASTN v2.12.0 with 80% identity and 1e-30 E-value cut-offs against the major VAGs in *Pseudomonas* spp. in virulence factor database (VFDB) of (http://www.mgc.ac.cn/cgi-bin/VFs/v5/main.cgi, accessed on 1 October 2022). To look for mobile genetic elements (MGEs), insertion sequence (IS) elements were searched using BLASTN v2.12.0 with 80% identity and 1e-30 E-value cut-offs against ISfinder database (https://isfinder.biotoul.fr/, accessed on 1 October 2022) [42], while integrons were predicted using integron_finder v2.0 (https://github.com/gem-pasteur/Integron_Finder, accessed on 1 October 2022) [43]. Furthermore, CRISPR-Cas regions were investigated using CRISPRCasFinder (https://crisprcas.i2bc.paris-saclay.fr/CrisprCasFinder/Index, accessed on 1 October 2022) [44], while bacteriophage sequences that integrated into our *P. aeruginosa* genomes were detected using the phage search tool enhanced release (PHASTER) (http://phaster.ca, accessed on 1 October 2022) [45,46]. Gene-encoding bacteriocins were explored using the bacteriocin genome mining tool (BAGEL4) (http://bagel4.molgenrug.nl/databases.php, accessed on 1 October 2022) [47]. 

### 4.6. Genomic Diversity and Phylogenetic Analyses

Pairwise average nucleotide identity (ANI) and pairwise single nucleotide polymorphism (SNP) distances were evaluated using FastANI v1.32 [48] and SNP-dists v0.8.2 (https://github.com/tseemann/snp-dists, accessed on 1 October 2022), respectively. Additionally, the pan-genome profiles of the studied 13 genomes were compared with 357 previously published genomes of *P. aeruginosa* from the National Center for Biotechnology Information (NCBI) database, using Roary v3.13.0 [49]. The metadata of the published genomes from several countries, including China, the USA, Germany, Japan, France, and Mexico [7,8,50,51,52,53], as well as other areas of Thailand [9,10] are exhibited in Table S8. A phylogenetic tree against a pan-genome matrix was generated using roary_plots (https://github.com/sanger-pathogens/Roary/tree/master/contrib/roary_plots, accessed on 1 October 2022). SNPs were called from core gene alignment using SNP-sites v2.4.1 [54]. Then, a core genome SNP-based phylogenetic tree was constructed using a geneious tree builder in Geneious R10.26 [36], with a selection of the neighbor-joining method and 500 bootstrap replicates. The circular tree was created using an online tool, the interactive tree of life (iTOL) (https://itol.embl.de/, accessed on 1 October 2022) [55].

## 5. Conclusions

This study exhibited antimicrobial resistance profiles and WGS data of the colonizing isolates of *P. aeruginosa* isolated from non-infected patients in a teaching hospital in Southern Thailand. Our findings revealed that all isolates were susceptible to many antimicrobial agents, except for colistin and carbapenems. Interestingly, a novel ST-3910 from the present study and 10 novel STs from other prior studies were assigned. The key genetic patterns, which might be used to predict their genetic evolution as well as their adaptation mechanism, were reported. Importantly, these data could probably be necessary as one of the important data points for tracking and managing the colonization and infection caused by this organism in the future.

## Figures and Tables

**Table 1:** Antimicrobial susceptibility in the colonizing isolates of *P. aeruginosa*.

Isolate Code	Minimum Inhibitory Concentration (µg/mL)										
	β-Lactam Combination Agents		Carbapenems			Lipopeptide	Aminoglycosides			Fluoroquinolones	
	TZP	C/T	IPM	MEM	DOR	CST	AMK	GEN	TOB	CIP	LVX
PA01	16 (S)	4 (S)	16 (R)	16 (R)	8 (R)	4 (R)	2 (S)	1 (S)	2 (S)	0.25 (S)	1 (S)
PA02	4 (S)	0.5 (S)	64 (R)	0.25 (S)	0.25 (S)	4 (R)	8 (S)	2 (S)	4 (S)	0.06 (S)	0.25 (S)
PA03	8 (S)	2 (S)	4 (I)	0.5 (S)	0.5 (S)	8 (R)	2 (S)	1 (S)	1 (S)	0.25 (S)	0.5 (S)
PA04	8 (S)	1 (S)	4 (I)	0.5 (S)	0.5 (S)	4 (R)	4 (S)	1 (S)	2 (S)	0.25 (S)	0.5 (S)
PA05	8 (S)	2 (S)	4 (I)	0.5 (S)	0.5 (S)	4 (R)	2 (S)	1 (S)	1 (S)	0.25 (S)	0.25 (S)
PA06	16 (S)	0.5 (S)	4 (I)	2 (S)	1 (S)	2 (I)	2 (S)	1 (S)	2 (S)	0.25 (S)	0.5 (S)
PA07	8 (S)	0.5 (S)	4 (I)	1 (S)	0.5 (S)	4 (R)	2 (S)	1 (S)	0.5 (S)	0.25 (S)	0.5 (S)
PA08	8 (S)	0.5 (S)	2 (S)	0.5 (S)	0.5(S)	2 (I)	2 (S)	1 (S)	1 (S)	0.25 (S)	0.5 (S)
PA09	8 (S)	1 (S)	1 (S)	0.5 (S)	0.5 (S)	2 (I)	2 (S)	1 (S)	1 (S)	0.25 (S)	0.5 (S)
PA10	8 (S)	2 (S)	2 (S)	0.5 (S)	0.5 (S)	4 (R)	4 (S)	1 (S)	2 (S)	0.25 (S)	0.5 (S)
PA11	16 (S)	0.5 (S)	2 (S)	0.5 (S)	0.5 (S)	2 (I)	2 (S)	1 (S)	1 (S)	0.25 (S)	0.25 (S)
PA13	8 (S)	0.5 (S)	32 (R)	8 (R)	2 (S)	4 (R)	4 (S)	1 (S)	2 (S)	0.25 (S)	0.5 (S)
PA14	16 (S)	1 (S)	4 (I)	0.5 (S)	0.5 (S)	8 (R)	2 (S)	1 (S)	0.5 (S)	0.25 (S)	0.5 (S)

S, susceptible; I, intermediate; R, resistant; TZP, piperacillin-tazobactam; C/T, ceftolozane-tazobactam; CST, colistin; IPM, imipenem; MEM, meropenem; DOR, doripenem; AMK, amikacin; GEN, gentamicin; TOB, tobramycin; CIP, ciprofloxacin; LVX, levofloxacin.

**Table 2:** Sequence types (STs) and serotypes of the colonizing isolates of *P. aeruginosa*.

Isolate Code	Patient’s Sex	Specimen	Serotype	Multilocus Sequence Typing (MLST)								Accession No.
				*acsA*	*aroE*	*guaA*	*mutL*	*nuoD*	*ppsA*	*trpE*	ST	
PA01	Female	Rectum	O6	17	5	6	3	4	15	10	647	JANTQX000000000
PA02	Male	Rectum	O11	20	30	64	26	30	24	32	3910 *	JANTQW000000000
PA03	Female	Rectum	O3	16	5	11	3	4	12	3	980	JANTQV000000000
PA04	Female	Throat	O6	6	5	1	30	4	6	27	1097	JANTQU000000000
PA05	Female	Rectum	O3	28	5	11	7	1	6	61	1240	JANTQT000000000
PA06	Male	Rectum	O10	5	8	119	6	12	6	3	1197	JANTQS000000000
PA07	Female	Throat	O11	5	4	5	5	5	20	4	532	JANTQR000000000
PA08	Female	Throat	O5	22	3	17	5	2	10	7	270	JANTQQ000000000
PA09	Male	Rectum	O6	11	57	7	3	4	15	1	500	JANTQP000000000
PA10	Male	Throat	O6	16	5	11	72	44	7	52	266	JANTQO000000000
PA11	Male	Throat	O1	47	8	7	6	8	11	40	313	JANTQN000000000
PA13	Male	Throat	O11	6	5	6	34	27	3	7	162	JANTQM000000000
PA14	Male	Throat	O11	6	5	6	34	27	3	7	162	JANTQL000000000

* Novel ST containing a new allelic profile.

**Table 3:** Clustered regularly interspaced short palindromic repeats and CRISPR-associated protein (CRISPR-Cas) system in the colonizing isolates of *P. aeruginosa*.

Isolate Code	ST	CRISPR-Cas				
		Region	Element	No. of Spacer	No. of *cas* Gene (Cas Type)	Direct Repeat (DR) Consensus/*cas* Gene
PA01	647	1	CRISPR	13		TTTCTTAGCTGCCTATACGGCAGTGAAC
		2	CRISPR	11		GTTCACTGCCGTATAGGCAGCTAAGAAA
		3	Cas cluster		6 (IF)	*cas6*, *csy3*, *csy2*, *csy1*, *cas3-cas2*, *cas1*
		4	CRISPR	7		TTTCTTAGCTGCCTACACGGCAGTGAAC
PA02	3910 *	1	CRISPR	10		GTTCACTGCCGTGTAGGCAGCTAAGAAA
		2	Cas cluster		6 (IF)	*cas1*, *cas3-cas2*, *csy1*, *csy2*, *csy3*, *cas6*
		3	CRISPR	4		TTTCTTAGCTGCCTATACGGCAGTGAAC
		4	CRISPR	7		TTTCTTAGCTGCCTATACGGCAGTGAAC
		5	CRISPR	27		CGGTTCATCCCCACGCATGTGGGGAACAC
		6	Cas cluster		8 (IE)	*cas2*, *cas1*, *cas6*, *cas5*, *cas7*, *cse2*, *cse1*, *cas3*
		7	CRISPR	4		CGGTTCATCCCCACACCCGTGGGGAACAC
		8	CRISPR	4		TTTCTTAGCTGCCTATACGGCAGTGAAC
		9	CRISPR	6		TTTCTTAGCTGCCTACACGGCAGTGAAC
		10	CRISPR	10		GTTCACTGCCGTGTAGGCAGCTAAGAAA
		11	CRISPR	5		TTTCTTAGCTGCCTATACGGCAGTGAAC
PA03	980	1	CRISPR	22		GTTCACTGCCGTATAGGCAGCTAAGAAA
		2	CRISPR	11		GTTCACTGCCGTGTAGGCAGCTAAGAAA
		3	Cas cluster		6 (IF)	*cas1*, *cas3-cas2*, *csy1*, *csy2*, *csy3*, *cas6*
		4	CRISPR	13		TTTCTTAGCTGCCTATACGGCAGTGAAC
PA04	1097	1	CRISPR	19		GTTCACTGCCGTGTAGGCAGCTAAGAAA
		2	Cas cluster		6 (IF)	*cas1*, *cas3-cas2*, *csy1*, *csy2*, *csy3*, *cas6*
		3	CRISPR	22		TTTCTTAGCTGCCTATACGGCAGTGAAC
PA05	1240	1	CRISPR	7		GTTCACTGCCGTATAGGCAGCTAAGAAA
		2	Cas cluster		6 (IF)	*cas6*, *csy3*, *csy2*, *csy1*, *cas3-cas2*, *cas1*
		3	CRISPR	14		TTTCTTAGCTGCCTACACGGCAGTGAAC
PA06	1197	ND	ND	ND		ND
PA07	532	1	CRISPR	11		GTGTTCCCCACGGGTGTGGGGATGAACCG
		2	Cas cluster		8 (IE)	*cas3*, *cse1*, *cse2*, *cas7*, *cas5*, *cas6*, *cas1*, *cas2*
		3	CRISPR	12		GTGTTCCCCACATGCGTGGGGATGAACCG
PA08	270	ND	ND	ND		ND
PA09	500	ND	ND	ND		ND
PA10	266	1	CRISPR	9		TTTCTTAGCTGCCTATACGGCAGTGAAC
PA11	313	ND	ND	ND		ND
PA13	162	ND	ND	ND		ND
PA14	162	ND	ND	ND		ND

ND, not detected. * Novel ST.

## Data Availability

The assembled genomes of all *P. aeruginosa* isolates in this study have been deposited in the NCBI GenBank under BioProject number PRJNA871949, with BioSample numbers SAMN30433208 to SAMN30433220.

## Supplementary Materials

The following supporting information can be downloaded at: https://www.mdpi.com/article/10.3390/antibiotics12010165/s1, Figure S1: BUSCO assessment results; Figure S2: Pan-genome matrix against phylogenetic tree based on accessory genes; Table S1: QUAST results of the colonizing isolates of *P. aeruginosa*; Table S2: Acquired antimicrobial resistance genes (ARGs) in the colonizing isolates of *P. aeruginosa*; Table S3: Insertion sequence (IS) elements in the colonizing isolates of *P. aeruginosa*; Table S4: Virulence-associated genes (VAGs) in the colonizing isolates of *P. aeruginosa*; Table S5: Bacteriocins in the colonizing isolates of *P. aeruginosa*; Table S6: CRISPR-Cas system in the colonizing isolates of *P. aeruginosa*; Table S7: Integrated bacteriophage genomes (IBGs) in the colonizing isolates of *P. aeruginosa*; Table S8: Metadata of previously published genomes from the NCBI database; Table S9: Pan-genome profiles of our genomes (*n* = 13) and previously published genomes (*n* = 357).
