# Supplementary figures and images for: Antimicrobial Susceptibility and Molecular Features of Colonizing Isolates of Pseudomonas aeruginosa and the Report of a Novel Sequence Type (ST) 3910 from Thailand
Source: Antibiotics (Basel). 2023 Jan 12;12(1):165. doi: 10.3390/antibiotics12010165 (PMC9854967; doi:10.3390/antibiotics12010165)

# BUSCO Assessment Results

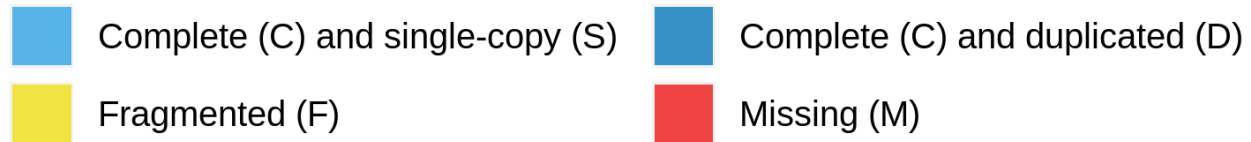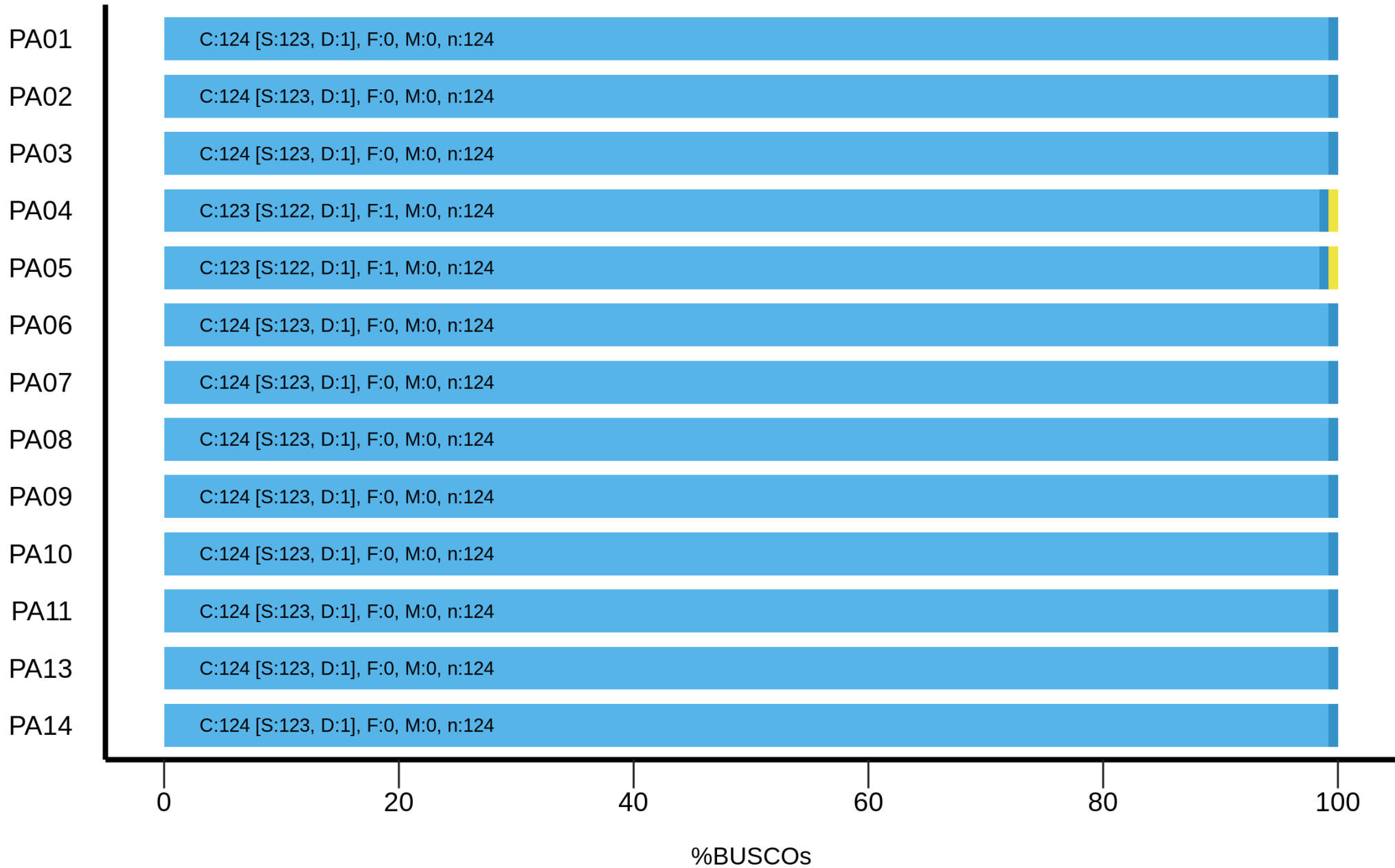

Supplement: Supplementary file 1 [file antibiotics-12-00165-s001.zip › Figure S1.pdf]

Tree  
(370 strains)

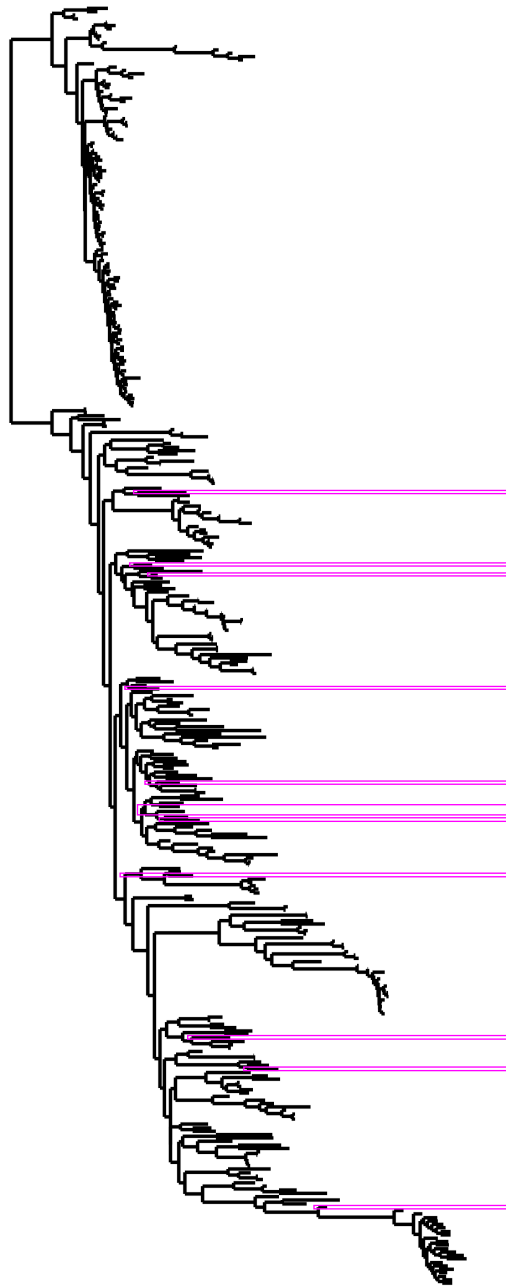

Roary matrix  
(35430 gene clusters)

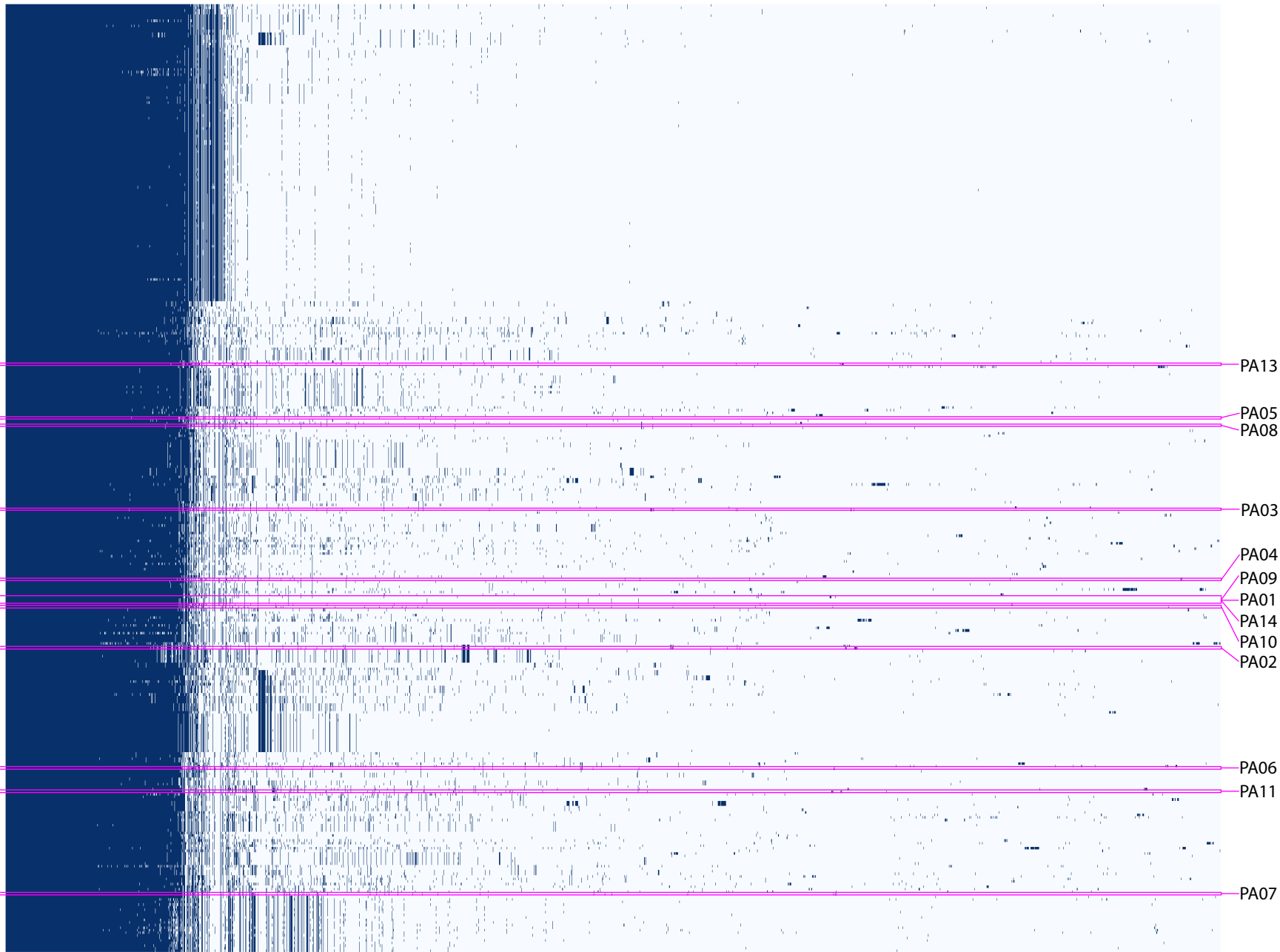

Supplement: Supplementary file 1 [file antibiotics-12-00165-s001.zip › Figure S2.pdf]
